# Effect of Co-exposure to Heat and Psychological Stressors on Sperm DNA and Semen Parameters

**DOI:** 10.1016/j.toxrep.2021.11.015

**Published:** 2021-11-25

**Authors:** Farnaz Abdollahi, Somayeh Farhang Dehghan, Saeid Amanpour, Abbas Haghparast, Siamak Sabour, Rezvan Zendehdel

**Affiliations:** a Department of Health and Safety at work, School of Public Health and Safety, Shahid Beheshti Medical Sciences, Tehran, Iran; b Environmental and Occupational Hazards Control Research Center, School of Public Health and Safety, Shahid Beheshti University of Medical Sciences, Tehran, Iran; c Cancer Biology Research Center, Cancer Institute of Iran, Tehran University of Medical Sciences, Tehran, Iran; d Neuroscience Research Center, Shahid Beheshti Medical Sciences, Tehran, Iran; e Department of Epidemiology, School of Public Health and Safety, Shahid Beheshti Medical Sciences, Tehran, Iran

**Keywords:** ANOVA, Analysis OF Variance, AO, Acridine Orange, BSA, Bovine Serum Albumin, CASA, Computer-Assisted Sperm Analysis, CPCSEA, Committee for the Purpose Of Control and Supervision Of Experiments on Animals, DNA, Deoxyribonucleic Acid, ROS, Reactive Oxygen Species, THI, Temperature Humidity Index, Heat Stress, Psychological Stress, Semen, Sperm DNA, Rat, Co-exposure

## Abstract

The present study aims to investigate the effects of co-exposure to heat and psychological stress on sperm DNA and semen parameters among male rats. The study was conducted on 40 healthy adult male Wistar rats. The rats were randomly categorized into four groups of same size consisting of a control group, a heat stress, psychological and co-exposure groups. The heat stress group was exposed to a temperature of 36 °C at 20% relative humidity. The psychological stress exposure group was subjected to three stressors including exposure to strobe light, noise and tilting cage. According the results,the co-exposure group had lower mean sperm parameters including sperm count (17.22 ± 4.22 10^6^/ml), motility (42.63 ± 12.95 %), viability (48.50 ± 23.25 %), normal morphology (56 ± 7.5%), progressive motility (11.61 ± 7.81%), non-progressive motility (31.18 ± 7.77%), curvilinear velocity (24.11 ± 3.81 μm/s) and straight-line velocity (3.2 ± 1.4 μm/s) when compared with those of the other groups (P = 0.001). Mean sperm immobility (57.36 ± 12.95%) and non-progressive motility (37.93 ± 11.15%) in the co-exposure group was higher compared to the other groups (P = 0.001 and P = 0.333, respectively). Assessment of damage to sperm DNA revealed that the heat exposure group had a higher percentage of sperm DNA damage (9.44 ± 6.80 %) compared to others (P = 0.185). In case of all of exposure scenario, the chance that the semen quality decreased compared to the control group has been increased. In general the combined stress had a greater significant effect on sperm parameters compared to other exposure groups, except for DNA damage.

## Highlights

• Co-exposure to heat and psychological stressors on semen quality have been studied.
• Combined exposure group had significantly lower semen quality compared with those of others.
• Heat exposure group had a higher percentage of sperm DNA damage compared to others.

## Graphical abstract

**Figure:**
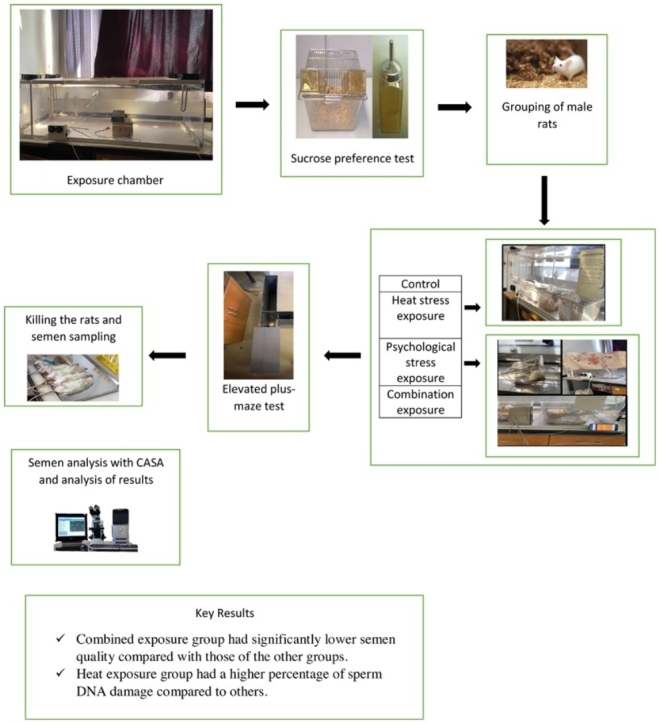

## 1 Introduction

A vital factor for survival among animals and humans is reproduction and procreation [1,2] There are reports of the effect of climate change on fertility rate decline [3]. Increased temperature at birth is also associated with adverse effects on fetal growth and longevity [4], as these are indicators of social health for the protection of generations and maintaining of population growth [5]. Numerous studies have been conducted on occupational exposure and its effects on the reproductive system [6,7]. Research has shown that half of the causes related to infertility among couples is due to problems associated with spermatogenesis [8]. Among the sensitive indices involved in the evaluation of the reproductive organs, spermatogenesis and sperm quality are perhaps the most notable. Evidence suggests a general reduction in sperm quality among men over the past 50 years [9,10].

Various different hazardous occupational and non-occupational factors effect human health [11,12]. Heat is an important environmental stressor in this regard [13]. A common problem for workers in occupational environments and especially in developing countries, is unfavorable weather conditions and working in warm environments [14]. Heat stress directly affects the health and performance of workers [15]. Increased body temperature has direct negative repercussions for the reproductive system in humans [13]. Sperm development among men is highly dependent on the temperature of the scrotum as it functions at 2.2 °C lower temperature than the rest of the body [14]. Research has shown that a deviation of 1 to 1.5 °C can cause changes in the functioning of the testes leading to disruption in spermatogenesis [16].

Cases of reduced sperm quality, sperm fertility and sperm motility along with abnormal sperm have been reported among men exposed to heat stress [5]. Research has also shown that heat stress exposure can significantly change semen parameters and can consequently result in changes in sperm morphology, sperm count and sperm motility [17]. Heat stress exposure has been observed to cause changes in sperm DNA [18]. Sperm cells that undergo apoptosis when exposed to heat have been observed to contain damaged DNA [19]. Exposure to heat causes increased spermatogenesis cell apoptosis which leads to the breakup of DNA and damages chromatin density [20].

Psychological stress is a personal experience which occurs when one is exposed to pressure or requests outside of one’s capabilities. An important cause of mental strain among workers is the stress associated with their job and occupation [21]. Stress induced by negative experiences in the work place can affect the behavior of workers [22]. Psychological stress can cause depression, anxiety, sexual disorders and infertility. This will also lead to reduced productivity and loss of profits for the employer as well as imposing medical costs on the individual [22,23]. Studies have shown that stress and anxiety increase oxidative stress resulting in higher amount of reactive oxygen species (ROS) and increased levels of cortisol [24]. Oxidative stress can influence both the quality of semen and sperm performance resulting in lower sperm motility and lower sperm fertility. Oxidative stress can also damage the chromatin within the sperm cell causing the breakup of DNA which results in the improper transfer of genes to the embryo and consequent birth defects [25]. Data suggests that psychological stress is involved in around half of the reported cases of male infertility [26]. Oxidative stress and anti-oxidant dis-equilibrium has a major role in the onset of various disorders including infertility [27,28].

Workers are exposed to hazardous physical agents (heat stress) and psychological stressors in their occupational environments which can affect their health and productivity. Usually when studying the health implications of exposure to these types of stressors, only one agent is regarded as the stressor, with few studies focusing on co-exposure to two or more stressors. However, workers are routinely exposed to multiple occupational stressors in their daily work schedule. As such, the present in-vivo study aims to investigate the effects of co-exposure to heat stress, as one of the most common types of hazardous physical occupational agents, and psychological stress, which has become common among workers due to working conditions, on the quality of sperm and sperm DNA among adult male rats.

## 2 Method

### 2.1 Study Sample

A total of 40 healthy adult male Wistar rats were used in the study. These rats weighed around 200 ± 75 grams and were 56 to 74 days old [29]. Using the Power and Sample Size Calculation software (v3.1.2), while allowing for a confidence interval of 95% in detecting 8 units of variance in the intended quantitative outcome (sperm concentration), along with a standard deviation of 3 units, suitable sample size was initially determined to be 5 rats per group [30]. This was later increased to 10 rats per group in order to account for future loss of samples during the study. A significance level below 0.05 was considered for the present study.

All sample rats were weighed in the initial stage of the study and were kept in separate cages equipped with temperature and humidity controls. A suitable light/dark cycle was provided throughout the study along with adequate food and water for the rats. These conditions consisted of a 12 -h dark/light cycle at 23 °C temperature and a humidity of 35% to 40% with a background noise level less than 35 dBA along with free access to rice bran pellets and water. These methods were approved by the ethics committee of the Shahid Beheshti University of Medical Sciences (ethics code IR.SBMU.PHNS.REC.1399.143). All protocols were carried out in accordance with the guidelines of the Committee for the Purpose of Control and Supervision of Experiments on Animals (CPCSEA).

### 2.2 Test Chamber

**Fig. 1:**
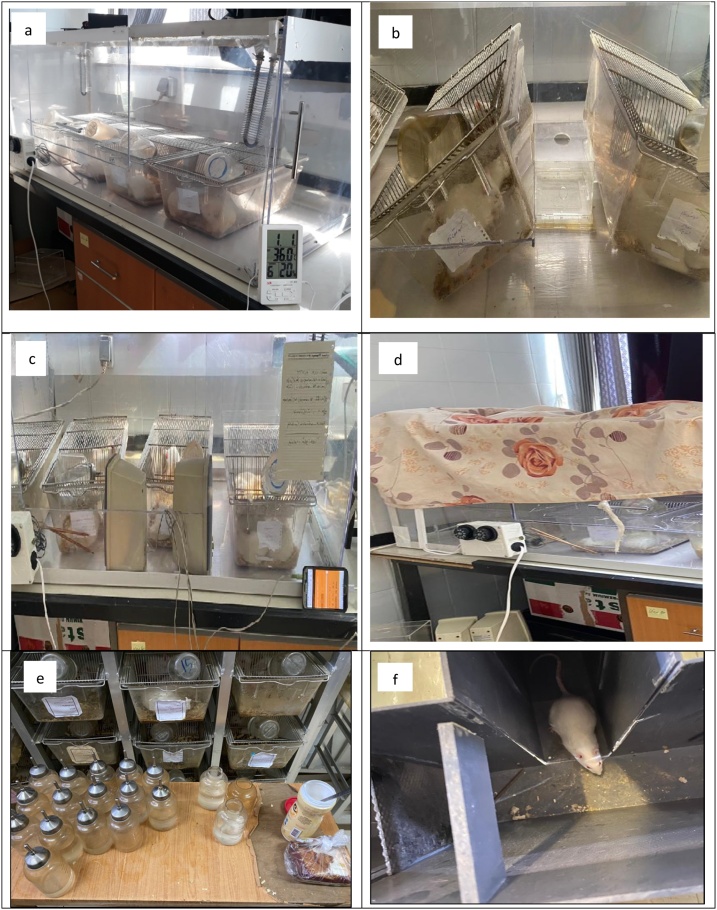
The subject rats being exposed to heat stress (a), tilting cage (b), ultrasonic noise (c) and strobe lighting (d) and also, the sucrose preference test (e) and elevated maze test (f).

A transparent Plexiglas chamber (150 × 50 × 50 cm) was made which could house 10 rats. This exposure chamber is capable of dynamically changing the flow of air while maintaining temperature and humidity at a constant level. The chamber is equipped with two heaters, a nebulizer and two ventilation fans. Psychological stress was induced using a strobe light (60 strobes per minute), a speaker and the Online Tone Generator software for producing ultra-sonic noise (Fig. 1) [31].

### 2.3 Exposure Conditions

The rats were randomly assigned into four groups of equal size with one group acting as the control and three others assigned as the exposure groups. The exposure groups consist of a heat stress exposure group, a physiological stress exposure group and a co-exposure group. The various exposure scenarios were carried out continuously over a period of 40 days. The order of exposure to the various psychological stressors was randomized each day. The specifics of the daily exposure conditions for each group is presented below:

• Heat stress exposure group: This group was exposed to 2 hours of heat stress per day (11 am to 13 pm) during the 40-day period. The intensity of heat stress was determined based on dry temperature and relative humidity as per the Temperature Humidity index (THI = 75.57 ± 3) which is an index used for evaluating heat stress among animals [32] (Fig. 1a).
• Psychological stress exposure group: This group was exposed to three different types of stressors including a strobe light (15 minutes per day), tilting the cage (15 minutes per day at a 45 ° angle) and noise (15 minutes per day at 80 dBA intensity with a frequency of 20 kHz) during the 40-day period. A minimum rest period of one-hour was administered between each exposure scenario. The order with which these stressors were exerted changed randomly each day during the 40-day period [33,34] (Fig. 1).
• Co-exposure (combined) group: This group was exposed to both heat stress (2 hour per day) and psychological stress (as per the protocols described above) including strobe light, noise and tilting the cage.
• Control group: This group was not exposed to any kind of stressor and were kept in comfortable conditions devoid of any heat or psychological stress.

Behavioral and psychological health was determined by administering the sucrose preference test before and after the exposure periods [34] (Fig. 1e). Additionally, at the 3^rd^, 5^th^ and 7^th^ day of exposure, three rats were selected at random and placed in an elevated maze test in order to determine the existence of psychological stress [35] (Fig. 1f).

### 2.4 Sperm Analysis

After the last day of exposure, the rats were euthanized and an incision was made in their abdomen with a sterilized scalpel. The tail of the Epididymis is severed from the left testicle and several incisions are made using a pair of scissors. The Epididymis tail is immediately placed inside a sterilized test tube containing 1 ml of T6 medium (containing 10 percent bovine serum albumin (BSA)) and then transferred to the lab. The samples were then placed in an incubator for 30 minutes at 37 °C and with 5% CO_2_ in order for the sperm to be extracted into the solution.

During sampling, sperm parameters such as sperm motility (progressive and non-progressive), sperm movement pattern (straight-line velocity and curvilinear velocity) and sperm concentration were evaluated using computer-assisted sperm analysis (CASA). This involves the use of a phase contrast microscope (NikonTM Eclipse E-200, Japan) connected to a camera (BaslerTM, A312FC, Germany) aided by software (SCA Microptic S.L., Spain) and using settings intended for rats. This was performed in the sperm biology lab of the Royan Institute.

The Eosin-Nigrosin staining method was used to count the number of live and dead sperm in order to determine sperm viability. The stained samples were observed using an optical microscope with at least 200 sperm being evaluated in each slide and the percentage of live sperm calculated.

Sperm morphology was evaluated via the SpermBlue stain method using a commercial stain kit (Microptic S.L., Spain) which includes a fixative and stain solution. This method is used to color all parts of the sperm (acrosome, head, midpiece and tail) with the basis for detection being the intensity of the color blue [36].

### 2.5 Sperm DNA Analysis

Sperm chromatin sensitivity to DNA damage was measured using the CASA device. This method is based on the metachromatic characteristic of a fluorescent material connected to DNA called Acridine Orange (AO) [37]. The sperm population with natural double stranded DNA had a green fluorescence (FL-1) which was considered as the largest sperm population. Those sperm cells with a more pronounced red florescence (FL-3) were situated to the right side of the main population which shows that these cells have undergone DNA denaturation.

### 2.6 Statistical Analysis

Statistical data analysis was done using SPSS v.22 (Chicago Il, USA). Descriptive results have been presented in the form of mean (standard deviation) and Median (interquartile range). Data distribution normality was determined using the Shapiro test. The Kruskal–Wallis test was used to compare median sperm count, sperm viability, sperm morphology, progressive and non-progressive motility among the four groups being studied. Then, the analysis of variance (ANOVA) test was performed in order to compare mean sperm motility, non-progressive motility, curvilinear velocity, sperm immobility, and the sperm DNA of the exposure groups with that of the control group. Dunnett's test was used to compare mean levels of 2 hormones in each exposure group with that of the control group. The parameter "B" of each exposure type on the target variable was determined using the univariate analysis of variance. A p-value lower than 0.05 was considered to be statistically significant.

## 3 Results

The mean weight of the rats on the control group, heat stress exposure group, psychological stress exposure group and the co-exposure group were 260 ± 20.2, 273 ± 16.2, 260 ± 22.3 and 275 ± 17.6 grams respectively (P > 0.05). The initial sucrose preference test revealed a mean consumption of 3.3 mg for plain water and 75.2 mg for the sucrose solution which results in an overall sucrose preference of 4.3. This shows that the rats were in a healthy mental state before the administration of the tests. The results of the elevated maze test performed on the psychological stress exposure group at the 10^th^, 25^th^ and 35^th^ day showed that the rats had spent 165, 190 and 210 seconds inside the covered arms respectively (total of 300 seconds). The results of the elevated maze test performed on the co-exposure group at the 10^th^, 25^th^ and 35^th^ day showed that the rats had spent 148, 220 and 246 seconds inside the covered arms respectively. This means that the rats in both these groups spent more time inside the covered areas and less time exploring uncovered areas which indicates the presence of mental stress.

**Fig. 2:**
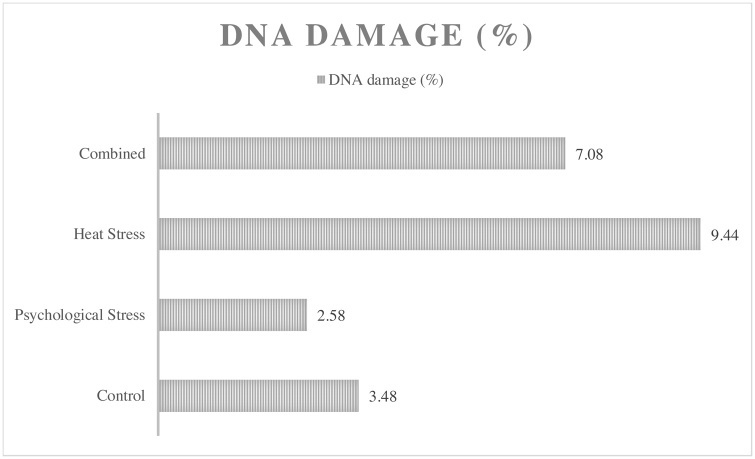
Comparison of mean DNA damage among different exposure group.

Assessment of semen quality (Table 1) revealed that the co-exposure group had lower mean sperm parameters including sperm count (17.22 ± 4.22 10^6^/ml), motility (42.63 ± 12.95 %), viability (48.50 ± 23.25 %), normal morphology (56 ± 7.5%), progressive motility (11.61 ± 7.81%), non-progressive motility (31.18 ± 7.77%), curvilinear velocity (24.11 ± 3.81 μm/s) and straight-line velocity (3.2 ± 1.4 μm/s) when compared with those of the other groups (P = 0.001). Mean sperm immobility (57.36 ± 12.95%) and non-progressive motility (37.93 ± 11.15%) in the co-exposure group was higher compared to the other groups (P = 0.001 and P = 0.333, respectively). Assessment of damage to sperm DNA revealed that the heat exposure group had a higher percentage of sperm DNA damage (9.44 ± 6.80 %) compared to others (Fig.2); however, no statistically significant difference in the percentage of sperm DNA damage was observed between them (P = 0.185).

**Table 1:** Results of sperm parameters among different exposure group

Group	Control		Psychological Stress		Heat Stress		Combined		P-value
Sperm Parameters	Mean (SD)	Median (IQR)	Mean (SD)	Median (IQR)	Mean (SD)	Median (IQR)	Mean (SD)	Median (IQR)	
Count (10^6^/ml)	37.88 (9.72)	40.58 (10.76)	24.69 (4.92)	23.74 (6.83)	24.31 (10.17)	26.73 (10.17)	15.29 (6.18)	17.22 (4.22)	0.001
Motility (%)	69.10 (8.94)	70.15 (9.78)	24.69 (4.92)	23.74 (6.83)	24.31 (10.17)	26.73 (10.17)	15.29 (6.18)	17.22 (4.22)	0.001
Viability (%)	76.30 (6.65)	79.0 (10)	59.50 (8.18)	61.0 (7.75)	59.30 (9.75)	60.5 (12.25)	49.6 (12.7)	48.50 (23.25)	0.001
Normal morphology (%)	78.70 (5.12)	78.50 (10.25)	65.5 (5.98)	65.0 (11)	66.0 (6.34)	65.5 (11.75)	56.4 (6.14)	56.0 (7.5)	0.001
Progressive motility (%)	33.44 (8.6)	35.25 (13.22)	18.7 (7.68)	19.75 (15.56)	19.07 (5.72)	20.48 (9.4)	12.04 (6.35)	11.61 (7.81)	0.001
Non-progressive motility (%)	35.66 (5.62)	35.10 (8.79)	34.10 (5.43)	34.76 (5.63)	35.41 (7.57)	37.11 (7.38)	31.18 (7.77)	37.93 (11.15)	0.333
Immobility (%)	31.09 (8.95)	29.84 (11.08)	49.19 (9.76)	49.29 (12.66)	45.51 (11.23)	44.56 (6.28)	57.36 (12.95)	57.40 (20.36)	0.001
Curvilinear velocity (μm/s)	47.16 (9.35)	43.21 (13.13)	28.95 (3.65)	30.45 (3.95)	31.92 (3.01)	30.92 (4.69)	24.11 (3.81)	24.90 (3.30)	0.001
Straight-line velocity (μm/s)	23.09 (5.42)	23.73 (9.12)	8.22 (3.84)	9.32 (7.34)	10.81 (4.27)	13.11 (8.36)	2.89 (1.3)	3.2 (1.4)	0.001

According to results of Dunnett's test (Table 2), a statistically significant difference was observed between the exposure groups and the control group for all measurable parameters (P < 0.05), except for non-progressive motility and DNA damage (P > 0.05). The combined group had the largest mean difference of sperm parameters with the control group among all exposure groups.

**Table 2:** A two-by-two comparison of sperm parameters in the control group and the exposure groups.

Group	Psychological Stress		Heat Stress		Combined	
Sperm Parameters	Mean difference	P-value	Mean difference	P-value	Mean difference	P-value
Count (10^6^/ml)	−13.19	0.002	−13.56	0..02	−22.59	0.001
Motility (%)	−18.29	0.002	−14.51	0.003	−26.46	0.001
Viability (%)	−16.80	0.001	−17.00	0.001	−26.70	0.001
Normal morphology (%)	−13.20	0.001	−12.70	0.001	−22.30	0.001
Progressive motility (%)	−14.73	0.001	−14.36	0.001	−21.39	0.001
Non-progressive motility (%)	−1.55	0.916	−0.25	1.000	−4.47	0.322
Immobility (%)	18.09	0.002	14.41	0.014	26.26	0.001
Curvilinear velocity (μm/s)	−18.20	0.001	−15.23	0.001	−23.04	0.001
Straight-line velocity (μm/s)	−14.87	0.001	−12.28	0.001	−20.19	0.001
DNA damage	−0.88	0.987	5.97	0.221	3.61	0.589

## 4 / 5000

Table 3 shows the results of univariate analysis of variance to determine the effect of the exposure scenario on sperm parameters. The co-exposure group had the highest parameter "B" (B = -22.59) on sperm count, so it can be said that in case of co-exposure to heat and psychological stressors, the chance that the sperm count decreases compared to the control group is 22 times. (P-value = 0.001). To determine the importance of each variable and their role in the regression model, the Standardized Coefficients column (Beta / B) should be considered. A variable with a larger standard coefficient will play a more effective role in predicting the dependent variable (sperm parameters). Moreover, the combined group had the highest parameter "B" on sperm motility (B = -26.46), viability (B = -26.70), normal morphology (B = -2.30), progressive motility (B = -2.39), non-progressive motility (B = -4.47), sperm immobility (B = 26.26), curved sperm motility (B = -23.04) and direct sperm motility (B = -20.19) compared to others exposure group. The effect of exposure scenario on sperm parameters was statistically significant, except for non-progressive motility and DNA damage. The psychological stress group had the higher parameter "B" on sperm parameters like motility, normal morphology, progressive motility, immobility, curvilinear velocity, straight-line velocity compared to heat stress group. However, the heat stress group had the higher B (B = 5.97) on DNA damage compared to psychological stress (B=-0.88) and combined groups (B = 3.61).

**Table 3:** The parameter "B" of exposure scenario on sperm parameters.

Group	Psychological Stress		Heat Stress		Combined	
Sperm Parameters	B	P-value	B	P-value	B	P-value
Count (10^6^/ml)	−13.19	0.001	−13.56	0.001	−22.59	0.001
Motility (%)	−18.29	0.001	−14.51	0.005	−26.46	0.001
Viability (%)	−16.80	0.001	−17.00	0.001	−26.70	0.001
Normal morphology (%)	−13.20	0.001	−12.70	0.001	−22.30	0.001
Progressive motility (%)	−14.73	0.001	−14.36	0.001	−21.39	0.001
Non-progressive motility (%)	−1.55	0.606	−0.25	0.934	−4.47	0.143
Immobility (%)	18.09	0.001	14.41	0.005	26.26	0.001
Curvilinear velocity (μm/s)	−18.20	0.001	−15.23	0.001	−23.04	0.001
Straight-line velocity (μm/s)	−14.87	0.001	−12.28	0.001	−20.19	0.001
DNA damage	−0.88	0.797	5.97	0.096	3.61	0.301

## 5 Discussion

The findings of the present study showed that co-exposure to heat and psychological stress caused reductions in sperm count, sperm motility, sperm viability, sperm morphology, progressive motility, non-progressive motility, curvilinear velocity and straight-line velocity among the subject rats, as well as an increased number of immotile sperm. Combined stress had a greater significant effect on sperm parameters compared to other exposure groups. Moreover, the both heat stress and psychological stress groups had the significant mean difference of sperm parameters with the control group. In case of all of exposure scenario, the chance that the semen quality decreased compared to the control group has been increased.

Previous studies have shown that heat stress can have a considerable effect on reproduction among animals [17]. Crespo et al. (2008) conducted a study to assess the scrotal heat stress effects on sperm viability, sperm DNA integrity, and the offspring sex ratio in mice. Their results showed that heat stress had led to reduced sperm concentration, sperm viability and sperm motility which is in agreement with the present findings [18]. Mahdivand et al. (2019) conducted a study aimed at the effects of heat stress on male rats. They found that considerable reduction in sperm concentration (p < 0.005), sperm count and sperm viability along with reduced fertility and increased chromatin irregularities in the heat stress exposure group [38]. Hamerezaee et al. (2018) also conducted a study evaluating semen quality among workers exposed to heat stress which showed that semen quality had decreased due to heat stress exposure [39] which agrees with the findings of the present study. The effects of heat stress on the reproductive system is mostly due to the destruction of testicular cells and reduced sperm quality. The heat stress can result in systemic physiological and biochemical changes in live organisms system, the hypothalamic–pituitary–adrenal axis and the hypothalamic-pituitary-testicular axis [40] and also I has been indicated that heat stress can lead to change in the neuroendocrine pathways in the sympathetic nervous group [41]. The negative effects on male reproductive indices such as Oligozoospermia, Azoospermia, Teratozoospermia have been reported from heat stress exposure [42,43]. The increased testicular temperature can cause the decreased spermatogenesis and normal sperm morphology. Usually, the temperature mechanism of the testes is capable of naturally maintaining the hypothermia of the scrotum, but in heat stress conditions, testes may be unable to effectively control scrotal temperatures which can lead to major changes in sperm characteristics [44,45]. Results from sperm DNA analysis shows co-exposure to heat and psychological stress has increased the percentage of DNA damage observed in the sperm of the rats. The co-exposure group had a larger B for DNA damage compared to both the control group and the psychological stress exposure group, while having a smaller parameter "B" (B = 5.97) compared to the heat stress exposure group. Research in the past decade has shown that the sperm of infertile men have higher amounts of DNA damage compared to those who are fertile [46]. Kaushik et al. (2019) looked at the effect of temperature on spermatogenesis disorders. Their results showed DNA breakup in all exposure groups which is in agreement with the present findings [47]. Hamilton et al. (2018) looked at the effects of heat stress on sperm DNA in adult rams and concluded that the heat stress exposure group had a higher level of DNA breakup [48] which is in agreement with the present findings.

Oxidative stress can also affect semen quality by reducing sperm motility and sperm fertility. During oxidative stress, the anti-oxidant defense mechanism is unable to neutralize excess free radicals resulting in their accumulation and subsequent disruption in the functioning of organs and the testes [25]. Zhang et al. (2020) conducted a study on the effects of psychological stress on reproductive indices. Analysis performed on semen after the rats were euthanized showed that psychological stress had reduced fertilization and increased cell apoptosis. Although sperm quality and sperm motility had also been reduced, improvements were seen 35 days after cessation of exposure which is in agreement with the present findings [49]. Arun et al. (2016) investigated protein changes in the testes of rats during spermatogenesis while undergoing chronic psychological stress. The results revealed an increase in sperm abnormalities and a decrease in sperm concentration in the exposure group compared to the control group with both being statistically meaningful (P < 0.05) [30]. These findings are in agreement with the present study. Psychological stress can lead to mental health issues such as depression and anxiety as well as sexual and reproductive disorder. The consequent increase in absentee days, lower productivity, increased costs and reduced profitability can also affect employers [22]. Ahmadi et al. showed that chronic exposure to noise can lead to changes in systolic and diastolic blood pressure, cognitive issues and even increased aggression [50]. Xiong et al. (2019) studied the reproductive performance of rats exposed to psychological stress caused by sudden loud noises. They foumd that the reduced sperm quality and increased sperm abnormalities with additional issues with gene expression, DNA structure and sperm cell apoptosis leading to infertility [51] which is also in agreement with the present findings.

Heat exposure has been known as a hazardous physical agent in occupational environments and can threaten the health and safety of personnel. This is especially a problem in developing countries and is most prominent in occupations such as casting, smelting, glassworks, ceramics, bakeries, and industrial scale kitchens and brick production. These occupations usually involve daily exposure to heat either due to the particular occupation or due to work being done in open air environments during warm seasons. Employees are affected by this heat stress and the results are observed as organ dysfunction. Research has shown that heat stress can affect the reproductive system in humans and animals, as was observed in the present study. The existence of psychological stressors in occupational environments as well as in the personal and social life of workers is also a problem which can, as a contributing background stressor, compound the negative physiological effects of heat stress. The results of the present study clearly show that co-exposure to heat and psychological stress can affect semen quality and cause DNA breakup. Preventive measures must be taken to reduce the negative effects of these stressors on the reproductive indices of personal at risk by reducing exposure to heat and controlling the level of psychological stress among workers.

There were some limiting factors for this study to draw confirmative conclusion on the co-exposure effect of heat stress and physiological stress on semen quality, including: maintaining the environmental conditions of the chamber, relatively small sample size, maintaining the dark conditions of the laboratory room in order to perform psychological stress (light strobe), and impossibility of repeating sample measurements due to a limited budget and ethical issues.

It is suggested that the studies be conducted with considering other environmental/occupational stressors such chemical (like heavy metals) and other physical hazardous agents (like electromagnetic fields) complying with psychological stress.

Author statement

All co-authors have seen and agree with the contents of the manuscript and we certify that there is no conflict of interest regarding the material discussed in the manuscript.

## Funding

This study was part of a research project supported by 10.13039/501100005851Shahid Beheshti University of Medical Sciences (Grant no. 25669)

## Ethics approval

Ethical approval for this study was obtained from School of Public Health & Neuroscience Research Center, Shahid Beheshti University of Medical Sciences (IR.SBMU.PHNS.REC.1399.144).

## Declaration of Competing Interest

The authors declare that they have no known competing financial interests or personal relationships that could have appeared to influence the work reported in this paper.

## Author Contribution

SFD, SA & AH contributed to the study design. SFD managed and planned the project. FA did the experiments. SFD, FA and SS were a major contributor in data analysis, interpretation and conclusion. All authors read and approved the final manuscript.

## Declaration of Competing Interest

The authors report no declarations of interest.
